# Adaptation of the classical end-point ITS-PCR for the diagnosis of avian trichomonosis to a real-time PCR reveals Bonelli’s eagle as a new host for *Trichomonas gypaetinii*

**DOI:** 10.1007/s00436-022-07693-3

**Published:** 2022-10-19

**Authors:** Sandra Alejandro Mateo, Iris Azami-Conesa, Bárbara Martín-Maldonado, Natalia Pastor-Tiburón, Raquel Martín-Hernández, Fernando González-González, María Teresa Gómez-Muñoz

**Affiliations:** 1 Department of Animal Health, Faculty of Veterinary Sciences, University Complutense of Madrid, Avenida Puerta de Hierro s/n, 28040 Madrid, Spain; 2 Group of Rehabilitation of the Autochtonous Fauna and Their Habitat (GREFA), Monte del Pilar, Majadahonda, 28220 Madrid, Spain; 3 Department of Veterinary Medicine, School of Biomedical and Health Sciences, Universidad Europea de Madrid, Villaviciosa de Odón, Spain; 4 Laboratorio de Patología Apícola, Centro de Investigación Apícola Y Agroambiental (CIAPA), Consejería de Agricultura de la Junta de Comunidades de Castilla-La Mancha, IRIAF. Instituto Regional de Investigación Y Desarrollo Agroalimentario Y Forestal, Camino de San Martín s/n, 19180 Marchamalo, Spain; 5 Instituto de Recursos Humanos Para La Ciencia Y La Tecnología (INCRECYT-FSE), Fundación Parque Científico Y Tecnológico de Castilla-La Mancha, 02006 Albacete, Spain

**Keywords:** Real time PCR, *Trichomonas gypaetinii*, *Trichomonas gallinae*, Sensitivity, Oropharyngeal trichomonosis, Avian trichomonosis

## Abstract

Avian trichomonosis is a parasitic disease caused mainly by *Trichomonas gallinae* and other *Trichomonas* species. It can be asymptomatic, or it can produce a necrotic lesion in the upper digestive tract and spread to other organs, causing the death of the infected birds. In this study, we aimed to evaluate an adapted real-time PCR method for the diagnosis of different genotypes and species of avian oropharyngeal trichomonads. Fifty-six samples from the oropharynx of Bonelli’s eagles (*Aquila fasciata*) obtained between 2018 and 2019 were analyzed using the real-time PCR and the end-point PCR, both targeting trichomonads ITS, and the results were compared by a coefficient of agreement. All positive samples were sequenced. The analysis showed a higher percentage of detection of real-time PCR ITS compared with end-point PCR ITS (64.3 vs 55.4%), and good agreement value (Kappa = 0.816). Melting temperature value for resulting amplicons of real-time PCR for avian trichomonads was 83.45 ± 0.72 °C. Genotypes A, D, and III were found among the sequences. Moreover, *Trichomonas gypaetinii*, a common species in scavenger birds, is reported for the first time in Bonelli’s eagles.

**Supplementary Information:**

The online version contains supplementary material available at 10.1007/s00436-022-07693-3.

## Introduction

*Trichomonas gallinae* (*T. gallinae,* Stabler, 1938) is a flagellated parasitic protozoan responsible for avian trichomonosis that may infect birds from different orders, especially Columbiformes, Falconiformes, Accipitriformes, Strigiformes, Psittaciformes, and Passeriformes (Forrester and Foster 2008). Columbiformes act as the main reservoir of the parasite, as it lives in their upper digestive tract, where it usually multiples by binary fission without producing many symptoms. In cases of highly virulent strains or immunosuppressed columbiform birds, symptoms may appear. Trophozoites damage the epithelial cells of the oropharynx, inducing the arrival of inflammatory cells (plasma cells and macrophages) and caseous-necrotic granulomas appear. Depending on the size of the lesions, the clinical signs comprise nasal discharge, sinusitis, dyspnea, dysphagia, regurgitation, sialorrhea, anorexia, poor body condition, cachexia, dehydration, bad appearance of plumage, lethargy, and difficulty to fly, and in severe cases, death by asphyxia, starvation, secondary bacterial infections, or dissemination through the body creating necrotic foci in several organs (Forrester and Foster 2008). Direct transmission to the fledglings occurs during feeding by their parents, while transmission between adults takes place during courtship or due to the consumption of contaminated water and carcasses (Amin et al. 2014).

Previous studies characterized isolates of *T. gallinae* from different avian species by PCR and subsequent genetic sequencing and described several genotypes of the intergenic spacer region ITS1-5.8S-ITS2 (ITS). Genotypes A and D, according to Gerhold et al. (2008), were predominant (Martínez-Herrero et al. 2014), and infection by the genotype A represented a risk factor for the development of the disease (Sansano-Maestre et al. 2009; Lawson et al. 2011; Chi et al. 2013; Martínez-Herrero et al. 2014; Martínez-Herrero et al. 2021). In addition, other genotypes of *Trichomonas* spp. have been isolated from several avian species. Some of them showed greater genetic homology with species such as *Trichomonas vaginalis*, *Trichomonas tenax* (Gerhold et al. 2008; Grabensteiner et al. 2010), *Tritrichomonas blagburni* (Girard et al. 2014), and *Trichomonas canistomae* (Martínez-Herrero et al. 2014). New species, such as *Trichomonas stableri* (Girard et al. 2013) and *Trichomonas gypaetinii* (Martínez-Díaz et al. 2015), have also been reported.

In the last decades, the consumption of pigeons by some species of birds of prey, like Bonelli’s eagles (*Aquila fasciata*), has increased, while in a parallel way, the incidence of the infection in these populations is also higher (Real et al. 2000; Palma et al. 2006). Raptors are more susceptible to the development of macroscopic lesions than columbiformes (Martínez-Herrero et al. 2014), in fact, trichomonosis is one of the concerns for the near-threatened species Bonelli’s eagle in the Mediterranean area of Europe (BirdLife International 2016). A high rate of *T. gallinae* infection was found in broods (41%), and macroscopic lesions at the oropharynx of birds ranged from 12.5 up to 87.5% of nestlings, depending on the year and the study (Hoefle et al. 2000; Real et al. 2000; Santos et al. 2019; Martínez-Herrero et al. 2021).

Culture is the only method by which the parasite can be isolated. However, the places where Bonelli’s eagle’s nests are located are difficult to access and this is the reason why the time from sampling to incubation of the cultures in the laboratory is usually too long, leading to false negatives frequently (Santos et al. 2019; Martínez-Herrero et al. 2021). Therefore, PCR from oropharyngeal swabs is preferable to obtain more reliable results (Gil-Sánchez et al. 2004; Martínez-Herrero et al. 2021).

The objective of our research was to adapt a real-time PCR method based on the ITS1-5.8S rRNA-ITS2 region (qPCR ITS) for the diagnosis of oropharyngeal avian trichomonosis. With this purpose, oropharyngeal samples from Bonelli’s eagle nestlings were employed, and the conditions for the qPCR ITS were set up, including the determination of the melting temperature (Tm) for the detection of avian trichomonads and calculation of the detection limit. The end-point PCR of the ITS1-5.8S rRNA-ITS2 region (end-point PCR ITS) method was also performed to compare the results obtained by each technique.

## Materials and methods

### Samples

Sampling was carried out during 2018 and 2019 as part of the conservation project AQUILA a-LIFE (LIFE 16 NAT/ES/000235). Sterile swabs were used to collect a total of 56 samples from the oropharynx of 54 Bonelli’s eagle chicks and 2 adults from different locations, including birds from natural nests from Spain and birds raised in recovery centers (GREFA, Majadahonda, Madrid, Spain; Center UFCS (LPO) in Vendée region, France). The two adults were wild birds born in Bulgaria and then transferred to GREFA facilities. The samples were stored at − 20 °C until used.

### DNA extraction

The cottons of the swabs were thawed at room temperature and then cut and introduced in Eppendorf tubes. DNA was extracted from the cottons using a commercial DNA extraction kit (NZY Tissue gDNA Isolation Kit; NZY Tech, Lisbon, Portugal) according to the manufacturers’ instructions, except the elution step in 50 µl instead of 100 µl. Positive (DNA from a culture of *T. gallinae* at stationary phase) and negative (sterile distilled water) samples were included in each batch, as previously described (Martínez-Herrero et al. 2021).

### End point PCR of the ITS1-5.8S rRNA-ITS2

The ITS1-5.8S rRNA-ITS2 region of the parasite was employed, as it is a highly sensitive target that presents tandem repeats and has shown greater sensitivity than other targets, such as 18S rRNA and Fe- Hydrogenase gene (Martínez-Herrero et al. 2021). The reaction was done in a final volume of 25 µl: 12.5 µl of a commercial kit (Supreme NZYTaq II 2 × green Master Mix; NZY Tech, Lisbon, Portugal), 8 µl of H_2_O, 1 µl of each primer, TFR1 10 µM (5’- TGCTTCAGTTCAGCGGGTCTTCC -3’) and TFR2 10 µM (5’-CGGTAGGTGAACCTGCCGTTGG-3’) (Felleisen 1997), and 2.5 µl of genomic DNA as template. Positive (DNA from *T. gallinae* isolate PM1/17, isolated in our lab from a wood pigeon) and non-template negative controls (NTC) were employed in each PCR set. The PCR protocol started with an initial step to activate the enzyme at 95 °C for 5 min, followed by 35 cycles of denaturation at 95 °C for 30 s, annealing at 68 °C for 30 s, extension at 72 °C for 15 s, and a final extension step at 72 °C for 10 min.

Electrophoresis was carried out in a 1.2% agarose gel stained with 10 µl GelRedTM Nucleic Acid Gel Stain 10,000X (Biotium, San Francisco, California, EEUU) at 90 V, 250 mA, and 300 W for 40 min. Five µl of each amplification product were loaded in each well, and 5 µl of NZYDNA Ladder III (0.2–10 kb) (NZY Tech, Lisbon, Portugal) were loaded in another well. Negative and positive controls were included in each set. The results were observed under ultraviolet light at 254 nm. Samples resulting in amplified fragments of 369 bp were considered positive (Felleisen 1997) and sent to sequence.

### Real-time PCR of the ITS1-5.8S rRNA-ITS2 region

The reaction was done in a final volume of 20 µl and contained 10 µl of a commercial kit (NZY Speedy qPCR Green Mastermix 2x, ROX, NZY Tech, Lisbon, Portugal) with Taq polymerase, reaction buffer, 3 mM MgCl2, dNTPs, and an intercalating dye (SYBR Green), 6.4 µl of H_2_O, 0.8 µl of each primer (TFR1 and TFR2) at 10 µM, and 2 µl of genomic DNA as template. The PCR protocol started with an initial step at 50 °C for 2 min, followed by a second step at 95 °C for 2 min and 40 cycles at 95 °C for 5 s and 60 °C for 20 s. A dissociation curve was performed by increasing the temperature from 60 to 95 °C for 5 s, followed by a step at 60 °C for 20 s and a final step at 95 °C for 5 s. Positive and negative controls were employed, likewise. The resulting curves were displayed by thermal cycler software QuantStudio 3 (Applied Biosystems, Foster City, California, USA). Since SYBR Green has non-specific affinity for double-stranded DNA, including primer dimers or contamination, and this fact cannot be differentiated by the amplification curves, the dissociation curves of positive controls were employed as a reference to classify the samples.

To test the specificity of the qPCR ITS, DNA samples from cultures of *Leishmania infantum* (reference strain MCAN/ES/98/LLM-722), *Trypanosoma cruzi* (clinical isolate Y, isolated in 1950 by Professor Pedreira da Freitas and donated by Dr. D. Miguel Belda Neto University of Araraquara (Brasil) in 1985), *Trichomonas vaginalis* (clinical isolate #1807, isolated and maintained in culture since 1995 and donated by Alexandra Ibañez Escribano from the Faculty of Pharmacy, UCM), and *Trichomitus* sp. (isolated from a Meller´s Chameleon and donated by Professor Francisco Ponce and Teresa Espinosa de los Monteros (Faculty of Pharmacy, UCM), were included as controls.

### Estimation of the detection limit

The detection limit of end-point PCR and qPCRs ITS was evaluated using 1/10 serial dilutions, up to six dilutions (1/10^−6^), starting with a sample of known concentration (23.5 µg/ml) of *T. gallinae* DNA, previously quantified by spectrophotometry at 260/280 nm (Eppendorf BioSpectrometer®; Eppendorf AG, Hamburg). The sample was obtained from 1 ml containing 309 × 10^3^ trophozoites of *T. gallinae* from a wood pigeon (isolate PM1-17) grown for 48 h in Trypticase-Yeast-Maltose (TYM) medium. The number of trophozoites/ml was determined by trypan blue staining in a Neubauer chamber. The highest dilution with a visible band on the agarose gel and the dilution with the highest detectable cycle threshold value (Ct) at the obtained Tm for the positive control were considered the detection limit for end-point PCR ITS and qPCR ITS, respectively.

### Statistical analysis

The end-point PCR and qPCR ITS methods were evaluated. The agreement between the two methods was determined by calculating coefficient Kappa value. All the parameters were calculated employing 95% confidence intervals using the tool WinEpi: Working in Epidemiology (www.winepi.net).

### Analysis of sequences

The amplification products of all positive samples from both PCRs ITS were purified using the commercial kit ExoSAP-IT (Exonuclease I/Shrimp Alkaline Phosphatase; Applied Biosystems, Foster City, California, EEUU) and sequenced in both directions at the Genomic Unit of the University Complutense of Madrid by a sanger sequencing. The chromatograms obtained from both directions were aligned and manually checked, and consensus sequences were obtained using Lasergene SeqMan software (DNASTAR, Madison, Wisconsin, USA). The consensus sequences were compared with previously published sequences available in the GenBank database using the BLAST algorithm (Basic Local Alignment Search Tool) of NCBI (https://blast.ncbi.nlm.nih.gov/), but only one of them not previously described in Bonelli’s eagle was submitted to GenBank (acc.n. MZ363736), since the rest of the sequences belonged to genotypes already described in this species.

### Ethics

Samples were taken as a part of the conservation project AQUILA a-LIFE (LIFE 16 NAT/ES/000235) for diagnosis purposes, under a non-invasive veterinary procedure. The research work is compliant with the national regulations on this subject.

## Results

### Estimation of the detection limit

End-point PCR ITS and qPCR ITS detected a positive result until 1/100 DNA dilution, corresponding to 588 pg of DNA (7.7 trophozoites) in end-point PCR ITS and 470 pg of DNA (equivalent to 6.2 trophozoites) in qPCR ITS, due to the different volume of sample employed in each method (2.5 and 2 µl, respectively).

In qPCR ITS (Supplementary Figs. 1 and 2), amplification occurs in earlier cycles at a higher concentration of DNA, as expected.

### End-point PCR and qPCR ITS results

No unspecific detection was observed in negative controls of both methods. End-point PCR ITS showed 31 positives out of 56 samples (55.4%), while qPCR ITS identified 36 positive samples (64.3%) (Table 1). All the samples with a positive result in end-point PCR ITS were also positive in qPCR ITS.

**Table 1:** Comparison of results obtained with end-point PCR ITS and qPCR ITS of *T. gallinae* with samples from the present study

qPCR ITS			
End-point PCR ITS	Positive	Negative	Total
Positive	31	0	31
Negative	5	20	25
Total	36	20	56

Melting temperature (Tm) values of the positive control varied between 83 and 83.9 °C (average Tm 83.45 °C). Tm values of positive samples ranged between 82.73 and 83.95 °C (average Tm 83.34 °C). Ct values ranged from 18.43–38.98. Most of the 36 positive samples at qPCR ITS displayed a Ct value lower than 35, except two, with Ct values of 36.05 and 38.98 and that rendered two sequences (Table 2). Therefore, the parameters selected to consider a sample as positive were established into Ct < 35 and Tm 83.5 ± 1 °C. According to this, only DNA of *Trichomonas vaginalis* was amplified (Tm value of 82.61, Ct value of 24.85), while the other protists DNA tested displayed a negative result, reinforcing the specificity of the methods for the genus *Trichomonas,* as referred in the original end-point PCR ITS (Felleisen 1997). In case of samples with Ct values higher than 35, the qPCR should be repeated and amplicons sequenced to confirm the results.

**Table 2:** Data from samples employed in this study, including results for end-point PCR and qPCR of the ITS region, and Tm values and CT values for qPCR ITS

Sample ID	Geographic location	Lesion	End-point PCR ITS and sequence type *	qPCR ITS and sequence type *	Tm value	CT value
19/1484	Madrid (Spain)	NO	( −)	( −)		
18/6719	Guadalajara (Spain)	NO	( +) D	( +) D	83.16 °C	30.49
18/6738	Bulgaria	YES	( +) D	( +) D	83.5 °C	32.05
18/6739	Bulgaria	NO	( +) D	( +) D	83.5 °C	31.18
19/0163	Madrid (Spain)	NO	( −)	( +) D	83.8 °C	38.98
19/1071	Center UFCS (France)	NO	( +) D	( +) D	83.5 °C	28.37
19/0459	Almería (Spain)	YES	( +) D	( +) D	83.5 °C	32.97
19/0331	Almería (Spain)	NO	( −)	( −)		
19/0411	Madrid (Spain)	NO	( −)	( −)		
19/0454	Almería (Spain)	NO	( −)	( −)		
19/0455	Almería (Spain)	NO	( +) D	( +) D	83.33 °C	22.46
19/0456	Almería (Spain)	NO	( +) D	( +) D	83.04 °C	22.42
19/0457	Almería (Spain)	NO	( +) mixed	( +) mixed	83 °C	27.27
19/0458	Almería (Spain)	NO	( +) D	( +) D	82.84 °C	31.65
19/0603	Madrid (Spain)	YES	( +) mixed	( +) mixed	83.5 °C	27.02
19/0151	Madrid (Spain)	YES	( +) A	( +) A	83 °C	30.58
19/0685	Málaga (Spain)	YES	( +) mixed	( +) A	83.04 °C	22.49
19/0686	Granada (Spain)	YES	( +) D	( +) D	83.19 °C	21.63
19/0688	Málaga (Spain)	YES	( −)	( −)		
19/0678	Málaga (Spain)	NO	( +) A	( +) A	83.35 °C	19.49
19/0679	Málaga (Spain)	NO	( −)	( +) D	83.16 °C	27.99
19/0680	Málaga (Spain)	YES	( +) A	( +) A	83.63 °C	26.18
19/0681	Málaga (Spain)	YES	( +) A	( +) A	83.61 °C	18.43
19/0689	Málaga (Spain)	NO	( −)	( −)		
19/0684	Málaga (Spain)	NO	( +) A	( +) A	83.5 °C	23.22
19/2785	Center UFCS (France)	NO	( −)	( −)		
19/2786	Center UFCS (France)	NO	( −)	( −)		
19/0838	Almería (Spain)	NO	( −)	( −)		
19/0839	Almería (Spain)	YES	( +) IIIG	( +) IIIG	83.66 °C	21.63
19/0840	Jaén (Spain)	NO	( +) D	( +) D	83.48 °C	20.16
19/0841	Jaén (Spain)	YES	( +) A	( +) A	83.62 °C	25.40
19/0976	Madrid (Spain)	NO	( +) mixed	( +) mixed	83.36 °C	36.05
19/2787	Center UFCS (France)	NO	( +) A	( +) A	83.63 °C	32.70
19/1019	Málaga (Spain)	NO	( +) A	( +) A	83.65 °C	20.78
19/1035	Madrid (Spain)	NO	( −)	( −)		
19/1104	Mallorca (Spain)	NO	( −)	( −)		
19/1069	Center UFCS (France)	NO	( −)	( +) mixed	83.95 °C	34.98
19/1072	Center UFCS (France)	NO	( −)	( −)		
19/1073	Center UFCS (France)	NO	( −)	( −)		
19/1074	Center UFCS (France)	NO	( −)	( +) D	83.77 °C	29.74
19/1070	Center UFCS (France)	NO	( −)	( −)		
19/2788	Center UFCS (France)	NO	( −)	( −)		
19/1468	Mallorca (Spain)	NO	( +) D	( +) D	83.03 °C	28.09
19/1378	Guadalajara (Spain)	YES	( −)	( +) D	83.04 °C	32.28
19/1379	Guadalajara (Spain)	YES	( −)	( −)		
19/1381	Toledo (Spain)	YES	( +) A	( +) A	83.77 °C	29.52
19/1638	Mallorca (Spain)	NO	( +) D	( +) D	83.7 °C	31.13
19/1639	Mallorca (Spain)	NO	( −)	( −)		
19/1570	Granada (Spain)	YES	( +) D	( +) D	82.73 °C	21.40
19/1637	Mallorca (Spain)	NO	( +) *T. gypaetinii*	( +) *T. gypaetinii*	82.74 °C	18.59
19/1788	Madrid (Spain)	NO	( −)	( −)		
19/1789	Madrid (Spain)	YES	( +) D	( +) D	83.17 °C	21.01
19/2861	Valencia (Spain)	YES	( −)	( −)		
19/0410	Mallorca (Spain)	NO	( +) mixed	( +) mixed	83 °C	27.39
17/1063	Mallorca (Spain)	NO	( −)	( −)		
19/1358	Mallorca (Spain)	YES	( +) IIIG	( +) IIIG	83.6 °C	28.64

^*^Sequence type A: EU215369, Sequence type D: EU215363, Sequence type IIIG: FN433473, *Trichomonas gypaetinii*: MZ363736 (this article). Mixed sequences correspond to A + D, according to the chromatogram information

### Agreement between end-point PCR ITS and qPCR ITS

The agreement between both diagnostic tests was good, according to the Cohen Kappa coefficient (Kappa = 0.816, 95% CI 0.558–1.173). A prevalence of 53.6% was observed in the sampled population using the end-point PCR ITS, a value similar to that obtained in previous years in the same bird species employing the same approach (30.8–52.8%) (Martínez-Herrero et al. 2021).

### Analysis of sequences

The sequences obtained from positive samples agreed in genotype using both PCRs, except one sample that displayed a mixed genotype in end-point PCR ITS and genotype A in qPCR ITS (Table 2). Five of the samples also showed double peaks in the chromatograms employing both methods, which is likely to correspond to mixed infections. Ten sample sequences with 100% homology with genotype A (Gerhold et al. 2008) (equivalent to ITS-OBT-Tg-1) (Martínez-Herrero et al. 2014), eighteen sample sequences showed 100% homology with genotype D (Gerhold et al. 2008) (equivalent to ITS-OBT-Tg-2) (Martínez-Herrero et al. 2014), two samples from nestlings showed 100% homology with genotype III (Grabensteiner et al. 2010) and one sample from a nestling (GenBank acc.n. MZ363736) presented 100% homology with *T. gypaetinii* (KM246602).

## Discussion

According to our results, both techniques present a similar detection limit. This similarity may be due to the enzymes used in both cases, which were highly sensitive and belonged to the same commercial company. Still, qPCR ITS detected a higher number of positives than end-point PCR. In comparative studies for other pathogens, qPCR has shown higher sensitivity compared to end-point PCR (Mohammadiha et al. 2013; Kim et al. 2014; Esvaran et al. 2019), which agrees with the results obtained in this study.

As recent studies have evidenced that *T. gallinae* is not the only species which can cause oropharyngeal trichomonosis, mixed infections including other trichomonads species were expected. ITS sequences from other genotypes and species had a similar size in base pairs, resulting in a positive band in agarose gel, and minor variations in Tm could be expected in the qPCR ITS, since the sequences vary slightly between different strains and species, and GC content determined melting temperatures, being higher in DNA with higher GC content than DNA with lower GC content. In previous studies based on the application of a qPCR method for the diagnosis of infections caused by other pathogens, such as *Candida* species, significant differences were found in Tm values due to a different composition in base pairs, which has allowed to distinguish between species belonging to the same genus (Asadzadeh et al. 2019). In our study, this fact was not observed, though, since three species of Trichomonads were amplified in a closed Tm value, including *Trichomonas vaginalis*. This fact has the advantage of allowing the identification of several genotypes or species present at the oropharynx of birds since mixed infections are common (Grabensteiner et al. 2010, Alrefaei et al. 2019).

Both, end-point PCR ITS and qPCR ITS are equally capable of correctly classifying non-infected animals, meaning that no false positive samples were obtained under our conditions since positive samples with both PCR methods displayed sequences of four genotypes from the family Trichomonadidae.

*Trichomonas gallinae* genotypes A and D are the two most frequent genotypes in avian species, as described in previous studies (Martínez-Herrero et al. 2014, 2021). Genotype III described in 2010 (Grabensteiner et al. 2010) has been found in columbiformes from Central Europe (Marx et al. 2017), and as a minor genotype of Bonelli´s eagles (Martínez-Herrero et al. 2021), probably because columbiform are an important part of the diet of the chicks, depending on the location on the nests.

*Trichomonas gypaetinii* was confirmed as a new species for the first time in 2015, mainly infecting vultures. Its sequence had greater genetic similarity (up to 97%) with *T. vaginalis* and *T. stableri* than with *T. gallinae* (Martínez-Díaz et al. 2015). So far, *T. gypaetinii* had only been found in bearded vultures (*Gypaetus barbatus*) (Grabensteiner et al. 2010), Egyptian vultures (*Neophron percnopterus*), black vultures (*Aegypius monachus*) (Martínez-herrero et al. 2014; Martínez-Díaz et al. 2015; Martínez-Herrero et al. 2019), a bald eagle (*Haliaeetus leucocephalus*) (Kelly-Clark et al. 2013), Steller’s sea eagles (*Haliaeetus pelagicus*), and white-tailed sea eagles (*Haliaeetus albicilla*) (Tomikawa et al. 2021) but never in Bonelli’s eagles. The Bonelli’s eagle nestling infected with *T. gypaetinii* was asymptomatic. According to the host range described so far for this parasite, its presence has been related to the necrophagy of mammal corpses, mainly ungulates. However, its presence in bald eagles, Steller’s sea eagles, and a Bonelli’s eagle is difficult to explain, since they are birds of prey that feed basically on fish, and occasionally on other birds in the first two eagle species, and leporids, partridges, and pigeons in the case of Bonelli’s eagles. This finding could be explained by the fact that maybe in the absence of preys, the diet of these birds included dead animal remains.

In this study, the differences in the composition of base pairs between the three genotypes of *T. gallinae* and *T. gypaetinii* sequences are not enough to allow the differentiation of the species/genotypes by comparison of the melting temperatures since they presented very similar values (82.73–83.48 °C in *T. gallinae* and 82.74 °C in *T. gypaetinii*). Similarly, in a recent assay using a qPCR method for the diagnosis of *T. gallinae*, in which a conserved region (18S rRNA) was selected for amplification, the technique was not able to discriminate between *T. gallinae* and *Tritrichomonas foetus* (Rentería-Solís et al. 2020). Another approach in the detection of trichomonads using qPCR was the one designed to differentiate *T. gallinae* from *Tetratrichomonas gallinarum*, but those species are more distant and probably with higher differences in GC content, and also, the last species is not common in the oropharynx of birds (Sigrist et al. 2022). An advantage of the use of ITS-PCR instead of 18S PCR could be a higher sensitivity, although this point should be further tested (Rentería-Solís et al. 2020; Martínez-Herrero et al. 2021).

In this study, we have adapted a qPCR technique to diagnose avian trichomonosis. The fact, that the qPCR ITS detected more positive samples than end-point PCR ITS suggests that it is a better choice for the monitoring of trichomonads infection. There are other advantages offered by this method, such as the avoiding of gels, the reduction of diagnostic times, and the possibility to semi-quantify parasitic DNA if Ct values were considered. However, according to the results obtained in this study, end-point PCR ITS would be a suitable alternative in those laboratories that do not have a real-time thermal cycler. Additionally, we report for the first time a Bonelli’s eagle nestling infected with *T. gypaetinii*, a species more related to scavenger birds, and occasionally found in other eagle species (Kelly-Clark et al. 2013; Tomikawa et al. 2021)*.*

## Supplementary Information

Below is the link to the electronic supplementary material.

Supplementary file1 (DOCX 237 kb)

Supplementary file2 (DOCX 164 kb)

## Data Availability

New sequences are deposited at GenBank.
