# Supplementary material for: Adaptation of the classical end-point ITS-PCR for the diagnosis of avian trichomonosis to a real-time PCR reveals Bonelli’s eagle as a new host for Trichomonas gypaetinii
Source: Parasitol Res. 2022 Oct 19;121(12):3663–70. doi: 10.1007/s00436-022-07693-3 (PMC9653314; doi:10.1007/s00436-022-07693-3)
Supplement: Supplementary file 1 — Supplementary file1 (DOCX 237 kb) [file 436_2022_7693_MOESM1_ESM.docx]

**Supplementary Fig. 1.** Representative melting curves corresponding to two negative controls (dark blue and grey at bottom), two positive controls (green and yellow), two positive samples (grey and blue) and one negative sample (red).

**
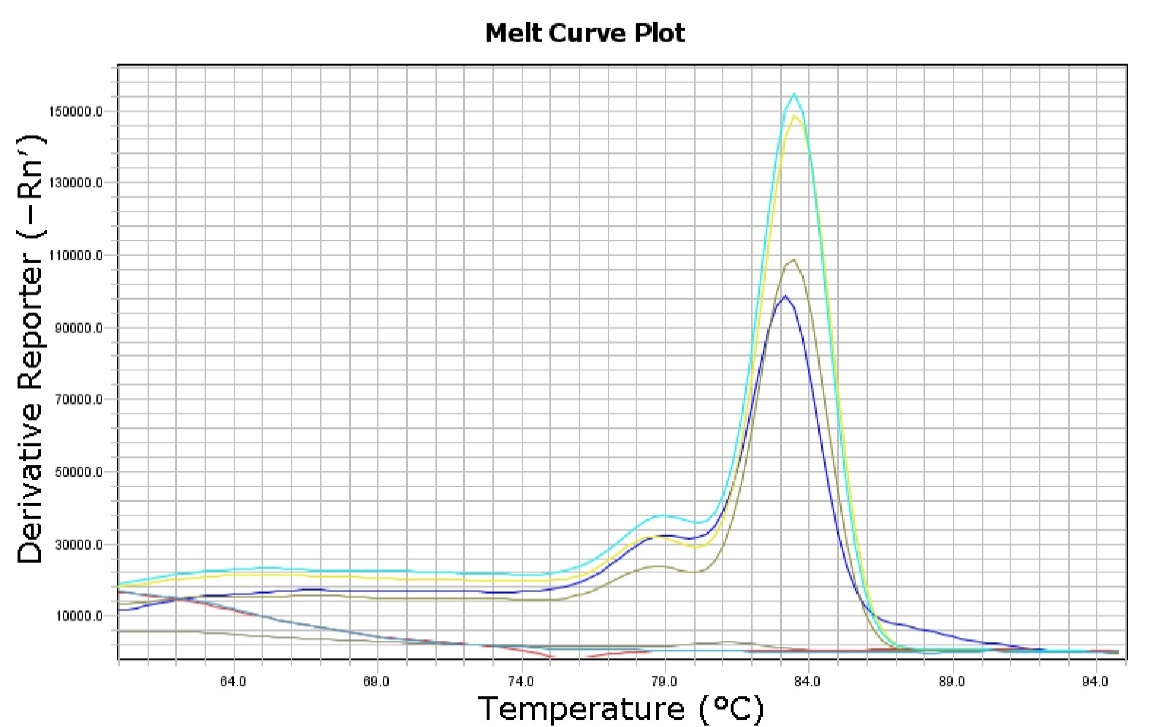
**

Article: Adaptation of the classical end-point ITS-PCR for the diagnosis of avian trichomonosis to a real-time PCR reveals Bonelli´s eagle as a new host for *Trichomonas gypaetinii*

By:

Sandra Alejandro Mateo ^1^, Iris Azami-Conesa ^1^, Bárbara Martín-Maldonado ^2^, Natalia Pastor-Tiburón ^2^, Raquel Martín-Hernández ^3.4^, Fernando González-González ^2^ and María Teresa Gómez-Muñoz ^1^ *

* mariateg@ucm.es
