# Supplementary material for: Adaptation of the classical end-point ITS-PCR for the diagnosis of avian trichomonosis to a real-time PCR reveals Bonelli’s eagle as a new host for Trichomonas gypaetinii
Source: Parasitol Res. 2022 Oct 19;121(12):3663–70. doi: 10.1007/s00436-022-07693-3 (PMC9653314; doi:10.1007/s00436-022-07693-3)
Supplement: Supplementary file 2 — Supplementary file2 (DOCX 164 kb) [file 436_2022_7693_MOESM2_ESM.docx]

**Supplementary Fig. 2.** Amplification curves corresponding to serial dilutions with a 1/10 dilution factor. From left to right: amplification curve of DNA without dilution, amplification curve of 1/10 dilution DNA, and amplification curve of 1/10^-2^dilution DNA. Dilutions from 1/10^-3^ to 1/10^-6^ are not shown since no amplification was detected.

**
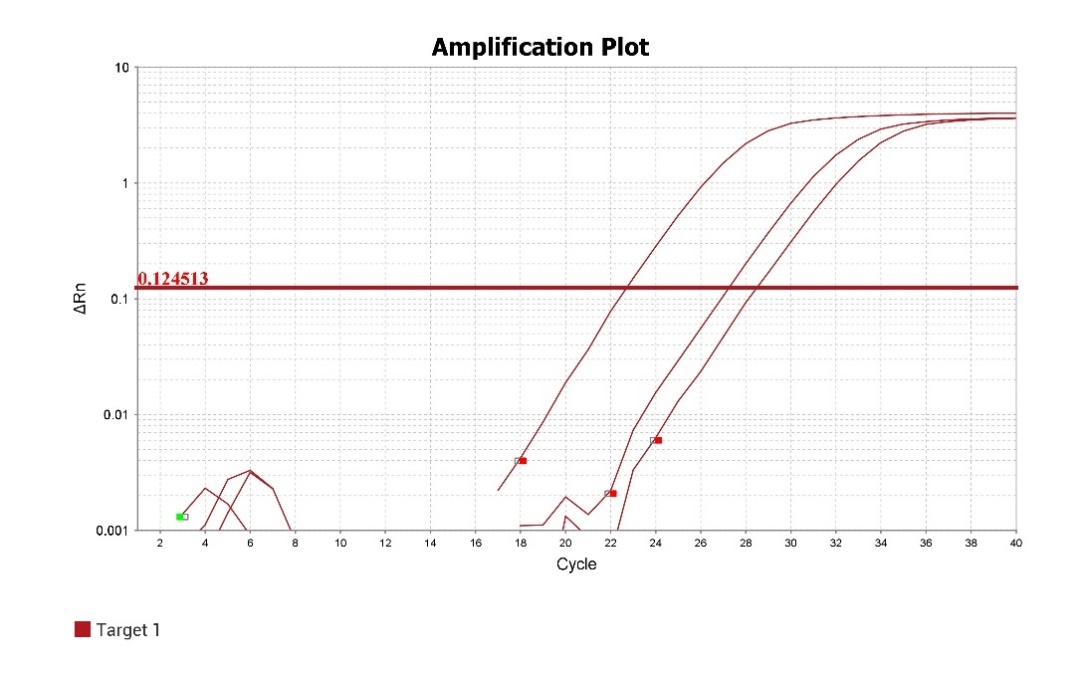
**

Article: Adaptation of the classical end-point ITS-PCR for the diagnosis of avian trichomonosis to a real-time PCR reveals Bonelli´s eagle as a new host for *Trichomonas gypaetinii*

By:

Sandra Alejandro Mateo ^1^, Iris Azami-Conesa ^1^, Bárbara Martín-Maldonado ^2^, Natalia Pastor-Tiburón ^2^, Raquel Martín-Hernández ^3.4^, Fernando González-González ^2^ and María Teresa Gómez-Muñoz ^1^ *

* mariateg@ucm.es
